# The Role of ANGPTL Gene Family Members in Hepatocellular Carcinoma

**DOI:** 10.1155/2022/1844352

**Published:** 2022-06-01

**Authors:** Yun Bai, Dan Lu, Di Qu, Yiwen Li, Ning Zhao, Guanghua Cui, Xue Li, Xiaoke Sun, Yanying Liu, Meiqi Wei, Yu Yang

**Affiliations:** Department of Oncology, Second Affiliated Hospital of Harbin Medical University, Harbin, China

## Abstract

**Background:**

Hepatocellular carcinoma (HCC) is highly aggressive with a poor prognosis and survival rate. Certain ANGPTL members have been implicated in tumor progression. However, the relevance of the ANGPTL gene family to HCC remains poorly understood. In this study, we explored the role of ANGPTLs in the prognosis of HCC.

**Methods:**

From the CCLE database, we studied the expression of ANGPTLs in a range of cancer cell lines. The UCSC, HCCDB, and Human Protein Atlas databases were used to analyze the differences in mRNA and protein expression of ANGPTLs in HCC tissues. Additionally, the correlation between ANGPTL mRNA and methylation levels and clinicopathological features were assessed in the TCGA database. The correlation between ANGPTL mRNA and overall survival was determined by the Kaplan-Meier plotter. cBioPortal database was used to analyze ANGPTL genomic alterations. Genes associated with ANGPTLs were determined by enrichment with KEGG. Moreover, the differentially expressed genes of ANGPTLs were analyzed by the LinkedOmics database, and the KEGG pathway and miRNA targets of ANGPTLs were also enriched.

**Results:**

There was a significant correlation between the ANGPTL members (excluding ANGPTL2) and the prognosis of HCC patients according to the Kaplan-Meier plotter analysis (*p* < 0.05). ANGPTL1 was the gene with the highest mutation frequency. ANGPTLs are involved in certain pathways that may influence the development of HCC.

**Conclusion:**

In summary, the expression of some members of ANGPTLs was significantly correlated with HCC prognosis, suggesting that the ANGPTL gene family members may be promising molecular markers for HCC treatment and prognosis.

## 1. Introduction

Hepatocellular carcinoma (HCC) is one of the most common malignancies in the world, accounting for 80% of primary liver cancer [1]. The morbidity and mortality rates have remained on the rise in recent years, with approximately 900,000 new cases of primary liver cancer and about 800,000 deaths from HCC worldwide, mostly in Southeast Asia [2]. Chronic hepatitis B virus infection has been shown to contribute to the development of HCC, whereas its acute insult could lead to fulminant viral hepatitis [3, 4]. Surgical treatments such as liver resection or liver transplantation are effective in HCC patients at an early stage. However, most patients are already in the advanced stages at the time of diagnosis and effective treatments are lacking [5]. Chemotherapy is the primary treatment for patients with advanced HCC; however, the highly aggressive and metastatic nature of HCC and resistance to chemotherapeutic agents lead to a poor prognosis [6, 7]. Therefore, there is a need to identify novel molecular markers that can show the therapeutic efficacy and predict the prognosis of HCC patients with high sensitivity and specificity.

Angiogenesis plays a vital role in the development of HCC [8]. Angiopoietin-like proteins (ANGPTLs) are similar to angiopoietins and include a family of eight members [9]. ANGPTLs consist of a coiled-coil structural domain (CCD) located at the amino (N) terminus of the protein and a fibrinogen-like structural domain (FLD) situated in the carboxyl (C) terminus of the protein [10], except ANGPTL8, which does not possess a C-terminal structure [11]. Multiple ANGPTLs are expressed in organs such as the liver, small intestine, vascular system, and hematopoietic system and play an essential role in regulating angiogenesis, inflammatory responses, and lipid metabolism [12, 13]. Recent studies have shown that ANGPTLs are involved in tumor progression, and their protein expression levels correlate with tumor invasion, angiogenesis, and metastasis [14, 15]. ANGPTLs can be expressed explicitly in specific cell types, suggesting that they may be critical in tumor development. Currently, no relevant studies have elucidated the role of the expression levels of the entire family of ANGPTLs in the prognosis of HCC patients. In this study, we employed online public database to explore the role and the prognostic value of ANGPTLs in HCC patients.

## 2. Materials and Methods

### 2.1. CCLE Database Analysis

The Cancer Cell Line Encyclopedia (CCLE) database, which has genomic and gene expression information of 1378 cell lines, enabled the analysis of RNA-seq data of ANGPTLs in liver cancer cell lines. Using R language (version 4.0.3), we compared the expression of the ANGPTL genes in HCC cells and other cell lines.

### 2.2. TCGA Database Analysis

The Cancer Genome Atlas (TCGA) collects clinical, genomic, transcriptomic, epigenomic, and proteomic data of 33 cancer types. UCSC Xena (http://xena.ucsc.edu/) is the portal site for the TCGA database. We downloaded RNA-seq data, clinical information data, and DNA methylation data of liver cancer samples (TCGA-LIHC) from the portal site UCSC Xena. We analyzed the adjacent normal tissues downstream as standard samples and defined the 2 KB region upstream and downstream of the transcription start site as the gene's promoter. The expression differences in ANGPTLs and in DNA methylation between tumor and normal tissues were compared using the rank-sum test function in R language (version 4.0.3). A significant correlation was determined by the Pearson correlation between DNA methylation levels of ANGPTLs and clinical characteristics (*p* < 0.05).

### 2.3. HCCDB Database Analysis

We analyzed the expression of ANGPTLs in 15 public datasets of HCC from the HCCDB database (http://lifeome.net/database/hccdb/home.html). Differential expression of ANGPTLs in HCC was analyzed using a *t*-test in R language defined as differentially expressed genes with corrected *p* values less than 0.001 and |log2FC| greater than 0.6 in at least 8 datasets.

### 2.4. Human Protein Atlas Analysis

The Human Protein Atlas (http://www.proteinatlas.org/) database collects information on proteins of interest to the ANGPTL1-5 gene. The protein expression levels of ANGPTL1-5 in tumor and normal tissues were compared.

### 2.5. Kaplan-Meier Plotter Analysis

The Kaplan-Meier plotter (http://kmplot.com/analysis/) assessed the impact of 54,000 genes in 21 cancer types on patient survival. We divided the patients into a high and a low expression group based on gene expression levels and compared their survival rates using the Kaplan-Meier survival plot. Risk ratios and log *p* values were calculated with 95% confidence intervals. The correlation between ANGPTL mRNA levels and overall survival in 364 HCC patients was analyzed using the Kaplan-Meier plotter, with *p* < 0.05 indicating statistical significance.

### 2.6. cBioPortal Analysis

The cBio Cancer Genomics Portal (https://www.cbioportal.org/) database was used to analyze the genomic alterations of ANGPTL family genes in HCC samples in TCGA (*n* = 372), including the expression of mutations, CNVs, and mRNAs.

### 2.7. STRING Database Analysis

The STRING database (https://string-db.org/) is a database for protein-interaction relationship searches. This database stores experimentally confirmed and predicted protein interaction information and can be used to construct protein-protein interaction networks. In this study, we used the STRING database to download the protein interaction information related to ANGPTLs and mapped the interaction network of ANGPTL interactions by Cytoscape 3.7 software, which can identify the essential genes interacting with ANGPTLs.

### 2.8. LinkedOmics Analysis

The LinkedOmics (http://www.linkedomics.org/login.php) website contains three analytic functions, LinkFinder, LinkInterpreter, and LinkCompare. In this study, we used the LinkFinder function to analyze ANGPTLs for the associated genes. Using the LinkInterpreter process, we considered FDR∗0.05 a significant association when comparing the Kyoto Encyclopedia of Genes and Genomes (KEGG) and miRNA-target enrichment analyses.

## 3. Results

### 3.1. CCLE Database Analysis of ANGPTL Expression in Different Cell Lines

**Figure 1:**
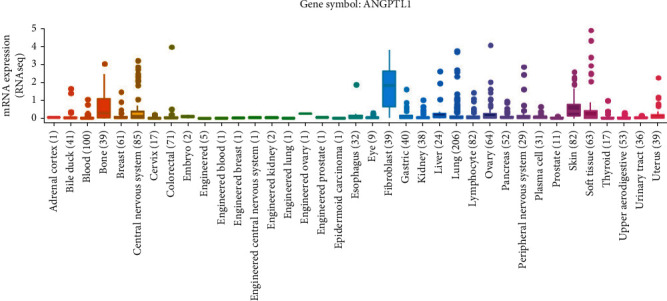
The mRNA expression levels of ANGPTL1 in several common cancer cell lines from the CCLE database.

We analyzed ANGPTL mRNA expression in several common cancer cell lines in the CCLE database. All HCC cell lines expressed the ANGPTL family of genes. There was a high protein expression of ANGPTL1, ANGPTL2, ANGPTL3, ANGPTL4, ANGPTL6, and ANGPTL8 in HCC cell lines (Figure 1 and Figure S1 A-G), indicating that ANGPTLs may play a vital function in HCC. We further found that ANGPTL1, ANGPTL3, ANGPTL4, and ANGPTL8 were significantly highly expressed in HCC cell lines than other types of cancer cell lines (*p* < 0.05, Figure S2 A-H).

### 3.2. Differential Expression of ANGPTLs in HCC and Adjacent Normal Tissues

**Figure 2:**
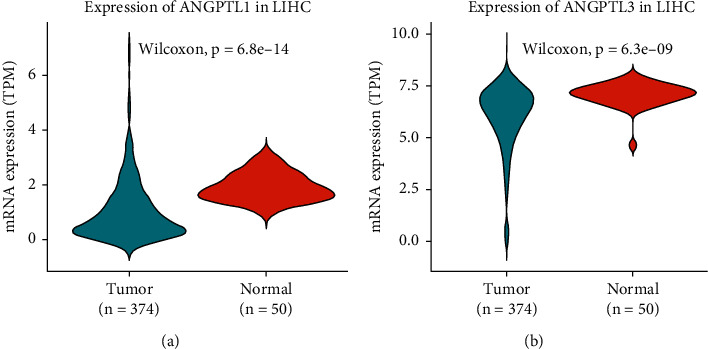
Relative expression of ANGPTLs in HCC samples and normal tissue samples from the UCSC database. Violin plot shows the expression of ANGPTL1 (a) and ANGPTL3 (b) mRNAs in HCC samples relative to normal samples, based on the TCGA database. LIHC: abbreviation for HCC of the liver from the TCGA database.

We downloaded expression data from the TCGA database for patients with HCC. The adjacent normal tissues were used as standard samples for downstream analysis. Using the rank-sum test, we examined whether tumor and normal tissues express ANGPTLs differently. We found that ANGPTL1 (Figure 2(a)), ANGPTL3 (Figure 2(b)), ANGPTL4 (Figure S3B), ANGPTL6 (Figure S3D), and ANGPTL7 (Figure S3E) were differentially expressed between tumor and normal tissues. ANGPTL2 (Figure S3A), ANGPTL5 (Figure S3C), and ANGPTL8 (Figure S3F) were similarly expressed between HCC and normal tissues.

### 3.3. HCCDB Database Analysis of ANGPTL Expression in HCC Cell Lines

**Figure 3:**
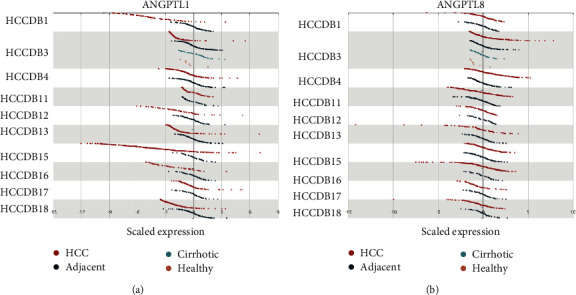
Relative expression of ANGPTLs in HCC samples, normal tissue, and cirrhotic and healthy samples in the HCCDB database. mRNA expression levels of ANGPTL1 (a) and ANGPTL8 (b) in different HCC datasets in the HCCDB database. Red: HCC samples; blue: adjacent normal tissue samples; cyan: cirrhotic samples; orange: healthy samples.

The expression of ANGPTLs was analyzed in the 15 HCC public datasets in the HCCDB database. The expression level of ANGPTL1 was low in HCC tissues from the HCCDB1, HCCDB12, HCCDB13, HCCDB15, HCCDB16, and HCCDB18 datasets (Figure 3(a)). The expression level of ANGPTL2 in HCC tissues was low in HCCDB13 and HCCDB15 (Figure S4A). The expression level of ANGPTL3 was low in HCC tissues in other datasets except for HCCDB4, HCCDB16, and HCCDB17 (Figure S4B). The HCCDB1, HCCDB3, HCCDB13, HCCDB15, HCCDB17, and HCCDB18 datasets showed that ANGPTL4 was downregulated in HCC tissues (Figure S4C). ANGPTL6 was downregulated in the HCC tissue in datasets other than HCCDB6 (Figure S4E). However, only ANGPTL8 was significantly highly expressed in HCC tissues from the HCCDB4 dataset (Figure 3(b)). Table S1 provides additional information.

### 3.4. Analysis of ANGPTL Gene Family Protein Expression Levels in HCC

**Figure 4:**
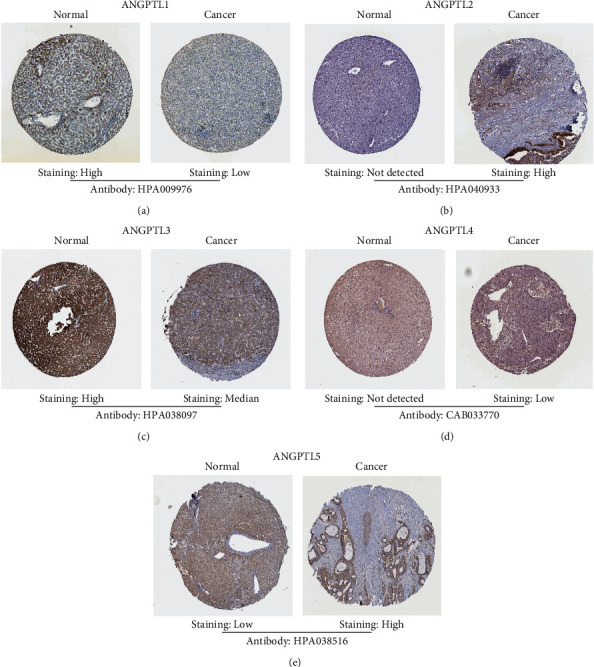
Comparison of protein levels of ANGPTL1 (a), ANGPTL2 (b), ANGPTL3 (c), ANGPTL4 (d), and ANGPTL5 (e) in HCC tissues with those of normal tissues by immunohistochemical staining.

We studied the immunohistochemical staining results of the ANGPTL gene family from the Human Protein Atlas database (Table 1). Some representative images from HCC tissues and normal tissues were shown (Figures 4(a)–4(e)). Normal tissues expressed ANGPTL1 and ANGPTL3, whereas tumor tissues expressed median and low levels. In contrast, ANGPTL2 and ANGPTL5 appeared highly expressed in tissues of HCCs. In comparison, ANGPTL4 was lower expressed in tumor tissues.

### 3.5. Association between ANGPTL Expression and Clinical Characteristics of HCC Patients

**Figure 5:**
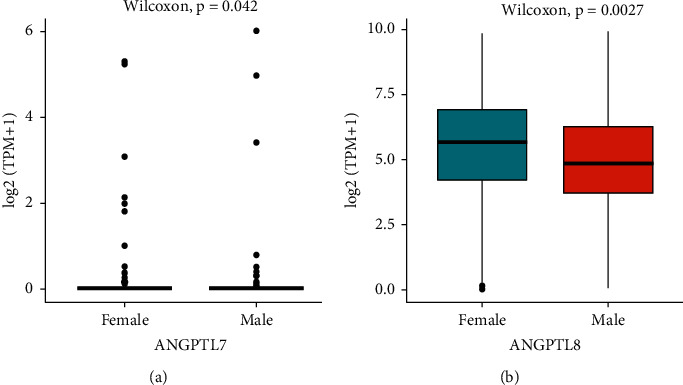
Correlation of ANGPTL7 (a) and ANGPTL8 (b) with gender based on TCGA database analysis.

We downloaded phenotypic data of LIHC from the TCGA database. The correlation of ANGPTL expression with age, gender, tumor stage, and tumor grade was further analyzed. The expression level of ANGPTL5 showed a significant negative correlation with age, ANGPTL5 and ANGPTL7 showed a significant positive correlation with tumor stage, and ANGPTL7 showed a significant negative correlation with tumor grade (Table 2). We examined differences in the expression levels of ANGPTLs by gender. Males had both high levels of ANGPTL7 and ANGPTL8 expression (Figures 5(a) and 5(b)). There was no significant difference in sex among the remaining ANGPTLs (Figure S5 A-F).

In addition, we analyzed the methylation levels of ANGPTLs in HCC tumor tissues versus normal tissues and their correlation with age, gender, tumor stage, and tumor grade. ANGPTL3 (Figure S6C), ANGPTL4 (Figure S6D), ANGPTL5 (Figure S6E), ANGPTL7 (Figure S6G), and ANGPTL8 (Figure S6H) were significantly hypermethylated in tumor tissues. A Pearson correlation analysis showed a significant positive correlation between DNA methylation levels of ANGPTL1 and ANGPTL4. In contrast, the DNA methylation levels of ANGPTL2, ANGPTL7, and ANGPTL8 were significantly positively correlated with tumor grade, and the DNA methylation levels of ANGPTL3, ANGPTL5, and ANGPTL7 were significantly negatively correlated with age (Table 3). The promoter regions of ANGPTL4, ANGPTL5, and ANGPTL6 genes showed hypermethylation in females, while ANGPTL8 showed hypomethylation in females (Figure S7 A-H).

### 3.6. Correlation between the Expression Levels of ANGPTLs and Overall Survival in HCC Patients

**Figure 6:**
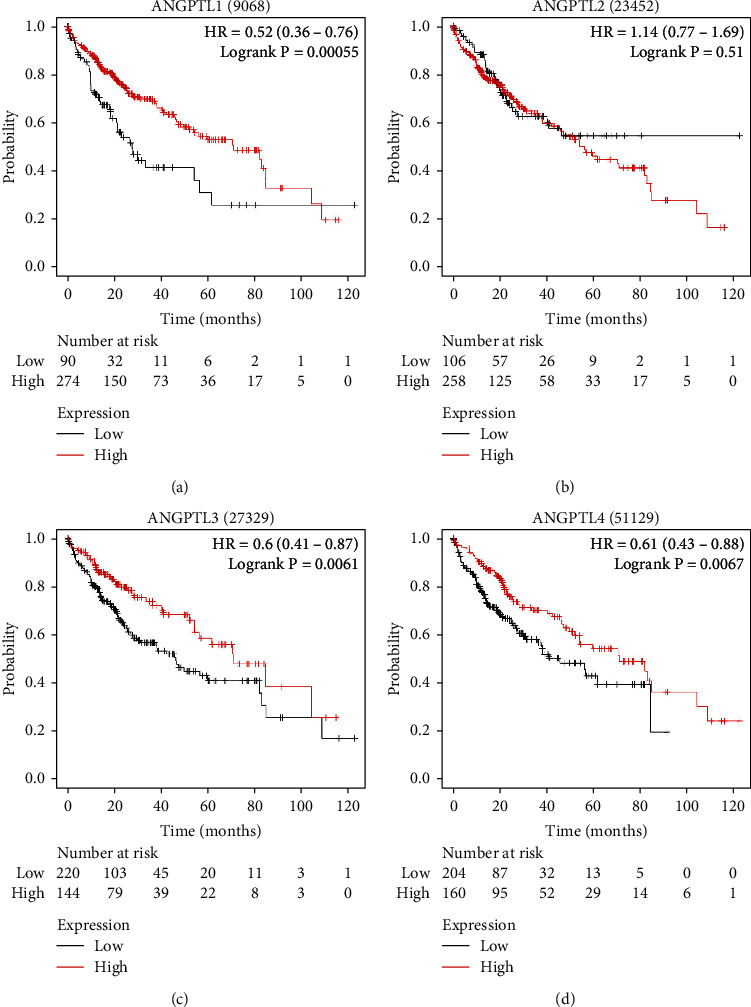
Association of expression of ANGPTLs with patient prognosis. The Kaplan-Meier plotter database was used to assess the correlation between the expression of ANGPTL1 (a), ANGPTL2 (b), ANGPTL3 (c), and ANGPTL4 (d) and the prognosis of HCC patients.

According to the Kaplan-Meier plotter, ANGPTL1 (Figure 6(a)), ANGPTL3 (Figure 6(c)), ANGPTL4 (Figure 6(d)), ANGPTL5 (Figure S8A), ANGPTL6 (Figure S8B), ANGPTL7 (Figure S8C), and ANGPTL8 (Figure S8D) were all associated with a good prognosis. There was no correlation between ANGPTL2 expression and survival of HCC patients (Figure 6(b)).

### 3.7. Genomic Alterations of ANGPTLs in HCC

**Figure 7:**
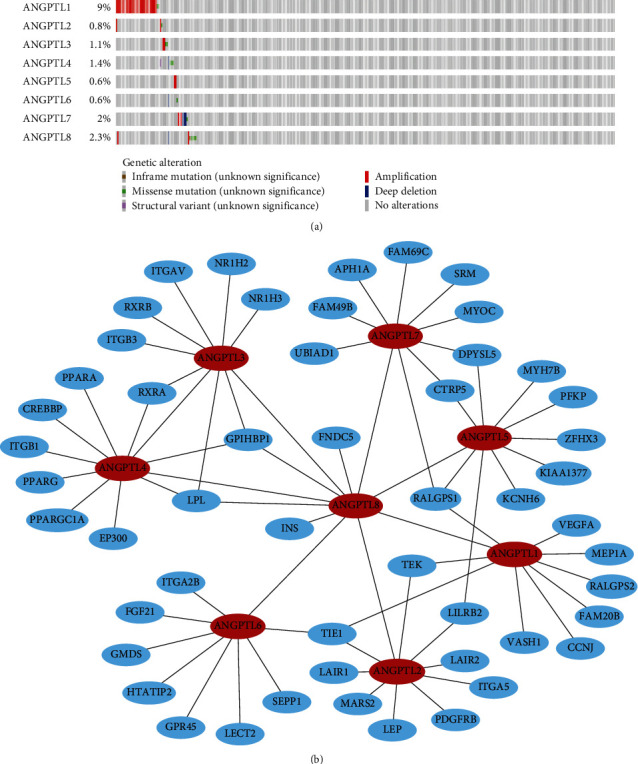
Gene mutation and biological interaction network of ANGPTLs in HCC. (a) Frequency and type of gene mutation of ANGPTLs in HCC patients from the TCGA database were analyzed by the cBioPortal database. Different colors show different types of gene alterations. (b) Interacting gene networks of ANGPTL gene families in HCC were collected through the STRING database. The red color represents seed genes and the blue color represents neighboring genes.

We examined mutation frequencies and types of the ANGPTL gene family in 372 TCGA-LIHC samples using the cBioPortal database (Figure 7(a)). ANGPTL1 had the highest mutation frequency, with 31 mutated samples and a mutation frequency of 9%. Other ANGPTL family members had lower mutation frequencies between 0.6% and 2.3% (Table S2). Then, we collected the genes with ANGPTL interactions from the STRING database and mapped the interaction network. GPIHBP1, LPL, TIE1, and RALGPS1 genes have regulatory relationships with multiple ANGPTL family members (Figure 7(b)).

Further, the mutation frequencies of ANGPTL family genes and neighboring genes were analyzed using the cBioPortal database. CYRIB (11%), APH1A (10%), and GPIHBP1 (10%) are all adjacent genes with high mutation rates. We downloaded copy number variation data of TCGA-LIHC from the UCSC Xena website and analyzed the copy number alterations of ANGPTL family genes and neighboring genes. UBIAD1, ANGPTL7, SRM, and FNDC5 had high copy number amplification, while APH1A, CYRIB, GPIHBP1, MYOC, GMDS, and VEGFA had high copy number deletion (Table S3).

### 3.8. KEGG Pathway Analysis of ANGPTL-Related Genes in HCC

**Figure 8:**
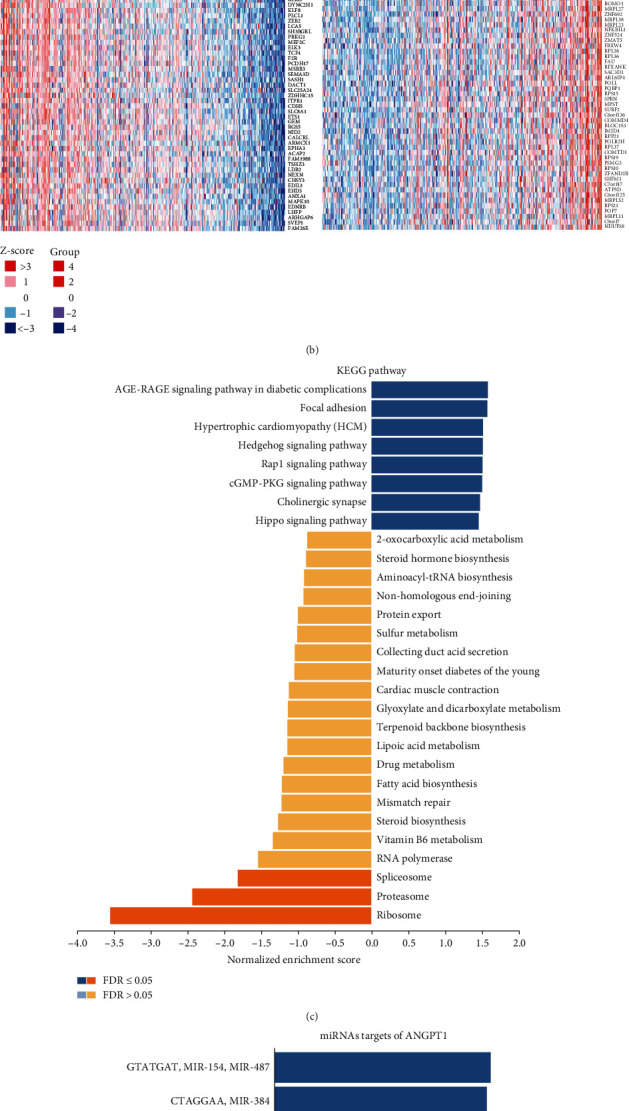
KEGG pathway enrichment analysis of ANGPTL1 coexpressed genes in HCC and miRNA targets of ANGPTL1. (a) Volcano plot showing differential expression of genes associated with ANGPTL1 in HCC and correlation analysis using Pearson. Green: negatively associated significant genes; red: positively associated significant genes. (b) Heat map showing the top 50 positively associated and top 50 negatively associated significant genes of ANGPTL1. (c) Analysis of KEGG pathway enrichment of ANGPTL1 coexpressed genes in HCC based on gene set enrichment analysis (GSEA). (d) miRNA of ANGPTL1 in HCC targets.

In this study, the previous analysis revealed that ANGPTL1 was significantly highly expressed in HCC and correlated with patient survival prognosis and had the highest mutation frequency in HCC. Consequently, we carried out additional analysis of the ANGPTL1 gene using the LinkFinder module in the LinkedOmics database, with 500 random perturbations. 8091 genes were significantly positively associated with ANGPTL1, and 2581 genes were significantly negatively associated (Figure 8(a)). The top 50 genes positively or negatively associated with ANGPTL1 were shown in the heat map of Figure 8(b). Similar heat maps of genes positively or negatively related to other ANGPTL family members are shown in Figures S9-S14. ANGPTL1 differentially expressed genes were enriched in ribosomes, proteasomes, and spliceosome pathways (Figure 8(c)). Genes related to other family members are also enriched in pathways associated with proteasome, ribosome, etc. (Figures S9-S14). To further explore the potential molecular mechanisms of ANGPTL1 in HCC, the enrichment of miRNAs targeting ANGPTL1, mainly miR-487, miR-384, miR-299-3P, miR-186, miR-23B, and miR527, was analyzed using the LinkInterpreter function in LinkedOmics (Figure 8(d)). In addition, miRNA target networks of other family members of ANGPTL were also analyzed, shown in Figures S9-S14.

## 4. Discussion

HCC is a malignancy characterized by aggressiveness and metastasis [16]. Early diagnosis is an urgent need for HCC patients, and the search for novel molecular markers that can predict HCC progression appears to be crucial. Tetrahydrobiopterin seems to be involved in the inhibition of HCC [17], and clinical scoring systems have been developed [18].

Studies have shown that ANGPTLs are significantly associated with tumor growth, metastasis, and drug resistance [19, 20]. To explore the role of ANGPTL expression levels in the prognosis HCC patients, we analyzed HCC patients using relevant online public database data. The results from the CCLE database analysis showed that the ANGPTL gene family was expressed in all HCC cell lines, as confirmed by previous studies. However, ANGPTL1 was found to be highly expressed in HCC cell lines. Previous studies showed that ANGPTL1 has angiogenesis-inhibiting and tumor-deactivating effects and belongs to tumor suppressors [10, 21, 22]. Recently, it was found that in HCC, ANGPTL1 promotes apoptosis by inhibiting the STAT3 pathway and reduces HCC cell activity by downregulating SLUG and SNAIL [23]. The HCCDB database revealed low ANGPTL1 expression in HCC tissues. According to the Human Protein Atlas database analysis, it agrees with the results of previous studies [24]. Analysis of the Human Protein Atlas database revealed that ANGPTL2 and ANGPTL5 were highly expressed in HCC tumor tissues, while ANGPTL4 was decreased. Studies have shown that ANGPTL2 is highly expressed in tumor tissues and has a procancer effect [25–28]. There are fewer reports about the correlation of ANGPTL5 in tumors [10, 22, 29]. Studies targeting the role of ANGPTL4 in tumors are more frequent compared to other members. Recent studies have shown that ANGPTL4 mRNA expression is significantly lower in HCC tissues than in nontumor tissues, consistent with the present study results [30]. However, other studies have also confirmed that ANGPTL4 seems to promote cancer development [31–33]. The function of ANGPTL4 in tumor tissues is still under debate.

From the TCGA database, we downloaded phenotypic data for LIHC. ANGPTLs are correlated with age, gender, tumor stage, and tumor grade in HCC patients. It has been reported that no significant correlation between ANGPTL family members and gender, except ANGPTL7 and ANGPTL8 [25, 26]. The relationship between ANGPTLs and the prognosis of HCC patients was analyzed using the Kaplan-Meier plotter. High expression of ANGPTL1 suggested an excellent prognosis. Chen H. et al. demonstrated a negative correlation between low expression of ANGPTL1 in tumor tissues and survival [34], which is consistent with the results of this study. In our study, high expression of ANGPTL8 predicts a good prognosis, whereas another study found that ANGPTL8 was overexpressed in HCC [35]. However, the biological function of ANGPTL8 in tumors has not been fully elucidated yet [10, 22, 36]. ANGPTL2 expression is associated with poor prognosis [37]. ANGPTL6 knockdown inhibits cancer cell metastasis [38]. Inconsistent with the results of the present study, further experimental verification is needed.

Using cBioPortal, we analyzed mutation frequencies and types of the ANGPTL gene family in 372 TCGA-LIHC samples. ANGPTL1 had a high mutation frequency of 9%. Further analysis of the mutation frequencies of ANGPTL family genes and neighboring genes revealed that CYRIB (11%), APH1A (10%), and GPIHBP1 (10%) had higher mutation frequencies. No study has investigated the role of the CYRIB gene in tumor progression. APH1A is involved in tumorigenesis and progression and plays a role in the invasion of cervical and pancreatic cancer [39, 40]. GPIHBP1 has a crucial role in lipid metabolism, and some studies have confirmed that GPIHBP1 is involved in the hepatic NF-*κ*B signaling pathway [41, 42]. These genes may be involved in the progression of HCC, and the inconsistent findings might be due to the difference in treatment [43].

## 5. Conclusion

In summary, we comprehensively analyzed HCC patient data through several online databases. The results suggest that ANGPTLs play a crucial role in HCC development, progression, metastasis, and prognosis.

## Figures and Tables

**Table 1:** Immunohistochemical staining results of ANGPTL protein obtained by Human Protein Atlas in normal liver tissues and HCC tissues.

Genes	Normal	Cancer (cases)				Antibody
		High	Median	Low	Not detected	
ANGPTL1	High	NA	NA	9	3	HPA009976
ANGPTL2	Not detected	2	2	NA	8	HPA040933
ANGPTL3	High	NA	4	2	5	HPA038097
ANGPTL4	Not detected	NA	NA	3	9	CAB033770
ANGPTL5	Low	1	2	1	8	HPA038516

**Table 2:** Correlation analysis between mRNA levels of ANGPTL family members and clinical characteristics.

Gene	Stage_R	Stage *p* value	Grade_R	Grade *p* value	Age_R	Age *p* value
ANGPTL1	-0.0475	0.3744	-0.0278	0.5937	0.0311	0.5415
ANGPTL2	0.0915	0.0865	-0.0332	0.5249	-0.0137	0.7874
ANGPTL3	-0.0626	0.2417	-0.0351	0.5014	0.0707	0.1643
ANGPTL4	-0.0411	0.4417	-0.0368	0.4801	-0.0598	0.2395
ANGPTL5	0.1374	0.0098	-0.0836	0.1083	-0.1226	0.0155
ANGPTL6	-0.0018	0.9725	-0.0979	0.0600	0.0776	0.1267
ANGPTL7	0.1195	0.0249	-0.1883	0.0003	0.0627	0.2170
ANGPTL8	0.0959	0.0725	0.0323	0.5351	-0.0610	0.2304

**Table 3:** Correlation analysis between DNA methylation of ANGPTL family members and clinical characteristics.

Gene	Stage_R	Stage *p* value	Grade_R	Grade *p* value	Age_R	Age *p* value
ANGPTL1	0.1077	0.0426	0.0487	0.3475	-0.0410	0.4177
ANGPTL2	0.0355	0.5047	0.1799	0.0005	-0.0097	0.8478
ANGPTL3	-0.0505	0.3424	0.0384	0.4588	-0.1525	0.0024
ANGPTL4	0.1529	0.0039	0.0477	0.3571	-0.0576	0.2543
ANGPTL5	0.0026	0.9606	0.0945	0.0678	-0.2306	3.83E-06
ANGPTL6	0.0521	0.3276	0.0269	0.6043	-0.0584	0.2480
ANGPTL7	-0.0028	0.9581	0.1613	0.0017	-0.1198	0.0175
ANGPTL8	0.0551	0.3009	0.1779	0.0005	-0.0471	0.3515

## Data Availability

The datasets generated during and/or analyzed during the current study are available from the corresponding author on reasonable request.

## Supplementary Materials

Supplementary Materials Table S1: differential expression of ANGPTL family members in HCC tissue in HCCDB database. Table S2: incidence rate of ANGPTL mutation in cBioPortal database. Table S3: the mutation incidence rate and copy changes of ANGPTL family genes and nearby genes. Figure S1: the mRNA expression levels of ANGPTL2 (A), ANGPTL3 (B), ANGPTL4 (C), ANGPTL5 (D), ANGPTL6 (E), ANGPTL7 (F), and ANGPTL8 (G), respectively, in several common cancer cell lines in the CCLE database. Figure S2: boxplot showing the difference in expression of ANGPTL mRNA levels in hepatocellular carcinoma cell lines versus other cancer cell lines. Figure S3: violin plot showing the relative expression of ANGPTLs in UCSC database in HCC samples and normal tissue samples. Panels (A)–(F) represent the expression of ANGPTL2, ANGPTL4, ANGPTL5, ANGPTL6, ANGPTL7, and ANGPTL8 mRNAs in HCC samples relative to normal samples based on the TCGA database. LIHC: abbreviation for hepatocellular carcinoma of the liver in the TCGA database. Figure S4: expression levels of ANGPTL2 (A), ANGPTL3 (B), ANGPTL4 (C), ANGPTL5 (D), ANGPTL6 (E), and ANGPTL7 (F) mRNAs in different HCC datasets in the HCCDB database were analyzed. Red: HCC samples; blue: adjacent normal tissue samples; cyan: cirrhotic samples; orange: healthy samples. Figure S5: correlation between ANGPTL gene family and sex-based on TCGA database. Panels (A)–(F) represent ANGPTL1, ANGPTL2, ANGPTL3, ANGPTL4, ANGPTL5, and ANGPTL6, respectively. Figure S6: analysis of ANGPTL DNA methylation levels in HCC tumor tissues versus normal tissues based on TCGA database. Panels (A)–(H) represent ANGPTL1, ANGPTL2, ANGPTL3, ANGPTL4, ANGPTL5, ANGPTL6, ANGPTL7, and ANGPTL8 DNA methylation expressions, respectively. Figure S7: analysis of the correlation between ANGPTL DNA methylation and clinical proportional characteristics by gender. Panels (A)–(H) represent the association of ANGPTL1, ANGPTL2, ANGPTL3, ANGPTL4, ANGPTL5, ANGPTL6, ANGPTL7, and ANGPTL8 DNA methylation with gender, respectively. Figure S8: association of expression of ANGPTLs with patient prognosis. The Kaplan-Meier plotter database was used to assess the correlation between the expression of ANGPTL5 (A), ANGPTL6 (B), ANGPTL7 (C), and ANGPTL8 (D) and the prognosis of HCC patients. Figure S9: KEGG pathway enrichment analysis of ANGPTL2 coexpressed genes in HCC and miRNA targets of ANGPTL2. (A) Volcano plot showing differential expression of genes associated with ANGPTL2 in HCC and correlation analysis using Pearson. Green: negatively associated significant genes; red: positively associated significant genes. (B) Heat map showing the top 50 positively associated and top 50 negatively associated significant genes of ANGPTL2. (C) Analysis of KEGG pathway enrichment of ANGPTL2 coexpressed genes in HCC based on gene set enrichment analysis (GSEA). (D) miRNA of ANGPTL2 in HCC targets. Figure S10: KEGG pathway enrichment analysis of ANGPTL3 coexpressed genes in HCC and miRNA targets of ANGPTL3. (A) Volcano plot showing differential expression of genes associated with ANGPTL3 in HCC and correlation analysis using Pearson. Green: negatively associated significant genes; red: positively associated significant genes. (B) Heat map showing the top 50 positively associated and top 50 negatively associated significant genes of ANGPTL3. (C) Analysis of KEGG pathway enrichment of ANGPTL3 coexpressed genes in HCC based on gene set enrichment analysis (GSEA). (D) miRNA of ANGPTL3 in HCC targets. Figure S11: KEGG pathway enrichment analysis of ANGPTL4 coexpressed genes in HCC and miRNA targets of ANGPTL4. (A) Volcano plot showing differential expression of genes associated with ANGPTL4 in HCC and correlation analysis using Pearson. Green: negatively associated significant genes; red: positively associated significant genes. (B) Heat map showing the top 50 positively associated and top 50 negatively associated significant genes of ANGPTL4. (C) Analysis of KEGG pathway enrichment of ANGPTL4 coexpressed genes in HCC based on gene set enrichment analysis (GSEA). (D) miRNAs of ANGPTL4 in HCC targets. Figure S12: KEGG pathway enrichment analysis of ANGPTL5 coexpressed genes in HCC and miRNA targets of ANGPTL5. (A) Volcano plot showing differential expression of genes associated with ANGPTL5 in HCC and correlation analysis using Pearson. Green: negatively associated significant genes; red: positively associated significant genes. (B) Heat map showing the top 50 positively associated and top 50 negatively associated significant genes of ANGPTL5. (C) Analysis of KEGG pathway enrichment of ANGPTL5 coexpressed genes in HCC based on gene set enrichment analysis (GSEA). (D) miRNA of ANGPTL5 in HCC targets. Figure S13: KEGG pathway enrichment analysis of ANGPTL6 coexpressed genes in HCC and miRNA targets of ANGPTL6. (A) Volcano plot showing differential expression of genes associated with ANGPTL6 in HCC and correlation analysis using Pearson. Green: negatively associated significant genes; red: positively associated significant genes. (B) Heat map showing the top 50 positively associated and top 50 negatively associated significant genes of ANGPTL6. (C) Analysis of KEGG pathway enrichment of ANGPTL6 coexpressed genes in HCC based on gene set enrichment analysis (GSEA). (D) miRNAs of ANGPTL6 in HCC targets. Figure S14: KEGG pathway enrichment analysis of ANGPTL7 coexpressed genes in HCC and miRNA targets of ANGPTL7. (A) Volcano plot showing differential expression of genes associated with ANGPTL7 in HCC and correlation analysis using Pearson. Green: negatively associated significant genes; red: positively associated significant genes. (B) Heat map showing the top 50 positively associated and top 50 negatively associated significant genes of ANGPTL7. (C) Analysis of KEGG pathway enrichment of ANGPTL7 coexpressed genes in HCC based on gene set enrichment analysis (GSEA). (D) miRNAs of ANGPTL7 in HCC targets.

## References

[1] Harris P. S., Hansen R. M., Gray M. E., Massoud O. I., McGuire B. M., Shoreibah M. G. (2019). Hepatocellular carcinoma surveillance: an evidence-based approach. *World Journal of Gastroenterology*.

[2] Sung H., Ferlay J., Siegel R. L. (2021). Global cancer statistics 2020: GLOBOCAN estimates of incidence and mortality worldwide for 36 cancers in 185 countries. *CA: a Cancer Journal for Clinicians*.

[3] Deng L., Li X., Shi Z., Jiang P., Chen D., Ma L. (2012). Maternal and perinatal outcome in cases of fulminant viral hepatitis in late pregnancy. *International Journal of Gynecology & Obstetrics*.

[4] Li X., Ma L., Yang Y., Shi Z., Zhou S. (2005). Prognostic factors of fulminant hepatitis in pregnancy. *Chinese Medical Journal*.

[5] Yang J. D., Hainaut P., Gores G. J., Amadou A., Plymoth A., Roberts L. R. (2019). A global view of hepatocellular carcinoma: trends, risk, prevention and management. *Nature Reviews. Gastroenterology & Hepatology*.

[6] Craig A. J., von Felden J., Garcia-Lezana T., Sarcognato S., Villanueva A. (2020). Tumour evolution in hepatocellular carcinoma. *Nature Reviews. Gastroenterology & Hepatology*.

[7] Woerns M. A., Weinmann A., Schuchmann M., Galle P. R. (2009). Systemic therapies in hepatocellular carcinoma. *Digestive Diseases*.

[8] Tanigawa N., Lu C., Mitsui T., Miura S. (1997). Quantitation of sinusoid-like vessels in hepatocellular carcinoma: its clinical and prognostic significance. *Hepatology*.

[9] Santulli G. (2014). Angiopoietin-like proteins: a comprehensive look. *Front Endocrinol (Lausanne)*.

[10] Carbone C., Piro G., Merz V. (2018). Angiopoietin-like proteins in angiogenesis, inflammation and cancer. *International Journal of Molecular Sciences*.

[11] Zeng L., Dai J., Ying K. (2003). Identification of a novel human angiopoietin-like gene expressed mainly in heart. *Journal of Human Genetics*.

[12] Abu-Farha M., Sriraman D., Cherian P. (2016). Circulating ANGPTL8/betatrophin is increased in obesity and reduced after exercise training. *PLoS One*.

[13] Arca M., Minicocci I., Maranghi M. (2013). The angiopoietin-like protein 3: a hepatokine with expanding role in metabolism. *Current Opinion in Lipidology*.

[14] Zhu P., Goh Y. Y., Chin H. F. A., Kersten S., Tan N. S. (2012). Angiopoietin-like 4: a decade of research. *Bioscience Reports*.

[15] Zhu P., Tan M. J., Huang R. L. (2011). Angiopoietin-like 4 protein elevates the prosurvival intracellular O_2_^−^:H_2_O_2_ ratio and confers anoikis resistance to tumors. *Cancer Cell*.

[16] Siegel R. L., Miller K. D., Jemal A. (2015). Cancer statistics, 2015. *CA: a Cancer Journal for Clinicians*.

[17] Vasquez-Vivar J., Shi Z., Tan S. (2022). Tetrahydrobiopterin in cell function and death mechanisms. *Antioxidants & Redox Signaling*.

[18] Yang Y., Deng L., Li X. (2012). Evaluation of the prognosis of fulminant viral hepatitis in late pregnancy by the MELD scoring system. *European Journal of Clinical Microbiology & Infectious Diseases*.

[19] Huang D., Sun G., Hao X. (2021). ANGPTL2-containing small extracellular vesicles from vascular endothelial cells accelerate leukemia progression. *The Journal of Clinical Investigation*.

[20] Monzavi N., Zargar S. J., Gheibi N., Azad M., Rahmani B. (2019). Angiopoietin-like protein 8 (betatrophin) may inhibit hepatocellular carcinoma through suppressing of the Wnt signaling pathway. *Iranian Journal of Basic Medical Sciences*.

[21] Sun R., Yang L., Hu Y. (2020). ANGPTL1 is a potential biomarker for differentiated thyroid cancer diagnosis and recurrence. *Oncology Letters*.

[22] Endo M. (2019). The roles of ANGPTL families in cancer progression. *Journal of UOEH*.

[23] Yan Q., Jiang L., Liu M. (2017). ANGPTL1 interacts with integrin *α*1*β*1 to suppress HCC angiogenesis and metastasis by inhibiting JAK2/STAT3 signaling. *Cancer Research*.

[24] Chen H. A., Kuo T. C., Tseng C. F. (2016). Angiopoietin-like protein 1 antagonizes MET receptor activity to repress sorafenib resistance and cancer stemness in hepatocellular carcinoma. *Hepatology*.

[25] Wang C., Tan R., Peng L., Zhang J. (2021). Relationship between miR-204 and ANGPTL2 expression and diagnosis, pathological stage, and prognosis in patients with colon cancer. *Translational Cancer Research*.

[26] Takeshita Y., Motohara T., Kadomatsu T. (2021). Angiopoietin-like protein 2 decreases peritoneal metastasis of ovarian cancer cells by suppressing anoikis resistance. *Biochemical and Biophysical Research Communications*.

[27] Wang X., Hu Z., Wang Z., Cui Y., Cui X. (2019). Angiopoietin-like protein 2 is an important facilitator of tumor proliferation, metastasis, angiogenesis and glycolysis in osteosarcoma. *American Journal of Translational Research*.

[28] Gao L., Ge C., Fang T. (2015). ANGPTL2 promotes tumor metastasis in hepatocellular carcinoma. *Journal of Gastroenterology and Hepatology*.

[29] Wang L., Geng T., Guo X. (2015). Co-expression of immunoglobulin-like transcript 4 and angiopoietin-like proteins in human non-small cell lung cancer. *Molecular Medicine Reports*.

[30] Ng K. T., Xu A., Cheng Q. (2014). Clinical relevance and therapeutic potential of angiopoietin-like protein 4 in hepatocellular carcinoma. *Molecular Cancer*.

[31] Kolb R., Kluz P., Tan Z. W. (2019). Obesity-associated inflammation promotes angiogenesis and breast cancer via angiopoietin-like 4. *Oncogene*.

[32] Amal S., Zidan H. E., Rashad N. M., Wadea F. M. (2017). Angiopoietin-like protein 3 and 4 expression 4 and their serum levels in hepatocellular carcinoma. *Cytokine*.

[33] Li H., Ge C., Zhao F. (2011). Hypoxia-inducible factor 1 alpha–activated angiopoietin-like protein 4 contributes to tumor metastasis via vascular cell adhesion molecule-1/integrin *β*1 signaling in human hepatocellular carcinoma. *Hepatology*.

[34] Chen H., Xiao Q., Hu Y. (2017). ANGPTL1 attenuates colorectal cancer metastasis by up-regulating microRNA-138. *Journal of Experimental & Clinical Cancer Research*.

[35] Dong X. Y., Pang X. W., Yu S. T. (2004). Identification of genes differentially expressed in human hepatocellular carcinoma by a modified suppression subtractive hybridization method. *International Journal of Cancer*.

[36] Tseng Y. H., Yeh Y. H., Chen W. J., Lin K. H. (2014). Emerging regulation and function of betatrophin. *International Journal of Molecular Sciences*.

[37] Thorin-Trescases N., Thorin E. (2017). High circulating levels of ANGPTL2: beyond a clinical marker of systemic inflammation. *Oxidative Medicine and Cellular Longevity*.

[38] Chen E., Tang C., Peng K., Cheng X., Wei Y., Liu T. (2019). ANGPTL6-mediated angiogenesis promotes alpha fetoprotein-producing gastric cancer progression. *Pathology, Research and Practice*.

[39] Yang S., Chen T., Huang L. (2019). High-risk human papillomavirus E7 maintains stemness via APH1B in cervical cancer stem-cell like cells. *Cancer Management and Research*.

[40] Jeon Y. H., Ha M., Kim S. W. (2019). Evaluation of the prognostic significances of *γ*-secretase genes in pancreatic cancer. *Oncology Letters*.

[41] Yu B., Zhang M., Chen J. (2019). Abnormality of hepatic triglyceride metabolism in Apc (Min/+) mice with colon cancer cachexia. *Life Sciences*.

[42] Allan C. M., Larsson M., Jung R. S. (2017). Mobility of "HSPG-bound" LPL explains how LPL is able to reach GPIHBP1 on capillaries. *Journal of Lipid Research*.

[43] Pan D., Si G., Du Y. Role of FOXQ1 in chemotherapy resistance of triple-negative breast ductal carcinoma in situ. *ISP Medicine*.

