# Supplementary material for: The Role of ANGPTL Gene Family Members in Hepatocellular Carcinoma
Source: Dis Markers. 2022 Jun 1;2022:1844352. doi: 10.1155/2022/1844352 (PMC9177307; doi:10.1155/2022/1844352)
Supplement: Supplementary Materials — Table S1: differential expression of ANGPTL family members in HCC tissue in HCCDB database. Table S2: incidence rate of ANGPTL mutation in cBioPortal database. Table S3: the mutation incidence rate and copy changes of ANGPTL family genes and nearby genes. Figure S1: the mRNA expression levels of ANGPTL2 (A), ANGPTL3 (B), ANGPTL4 (C), ANGPTL5 (D), ANGPTL6 (E), ANGPTL7 (F), and ANGPTL8 (G), respectively, in several common cancer cell lines in the CCLE database. Figure S2: boxplot showing the difference in expression of ANGPTL mRNA levels in hepatocellular carcinoma cell lines versus other cancer cell lines. Figure S3: violin plot showing the relative expression of ANGPTLs in UCSC database in HCC samples and normal tissue samples. Panels (A)–(F) represent the expression of ANGPTL2, ANGPTL4, ANGPTL5, ANGPTL6, ANGPTL7, and ANGPTL8 mRNAs in HCC samples relative to normal samples based on the TCGA database. LIHC: abbreviation for hepatocellular carcinoma of the liver in the TCGA database. Figure S4: expression levels of ANGPTL2 (A), ANGPTL3 (B), ANGPTL4 (C), ANGPTL5 (D), ANGPTL6 (E), and ANGPTL7 (F) mRNAs in different HCC datasets in the HCCDB database were analyzed. Red: HCC samples; blue: adjacent normal tissue samples; cyan: cirrhotic samples; orange: healthy samples. Figure S5: correlation between ANGPTL gene family and sex-based on TCGA database. Panels (A)–(F) represent ANGPTL1, ANGPTL2, ANGPTL3, ANGPTL4, ANGPTL5, and ANGPTL6, respectively. Figure S6: analysis of ANGPTL DNA methylation levels in HCC tumor tissues versus normal tissues based on TCGA database. Panels (A)–(H) represent ANGPTL1, ANGPTL2, ANGPTL3, ANGPTL4, ANGPTL5, ANGPTL6, ANGPTL7, and ANGPTL8 DNA methylation expressions, respectively. Figure S7: analysis of the correlation between ANGPTL DNA methylation and clinical proportional characteristics by gender. Panels (A)–(H) represent the association of ANGPTL1, ANGPTL2, ANGPTL3, ANGPTL4, ANGPTL5, ANGPTL6, ANGPTL7, and ANGPTL8 DNA methylation with gender, re [file 1844352.f1.zip › Figure S10.pdf]

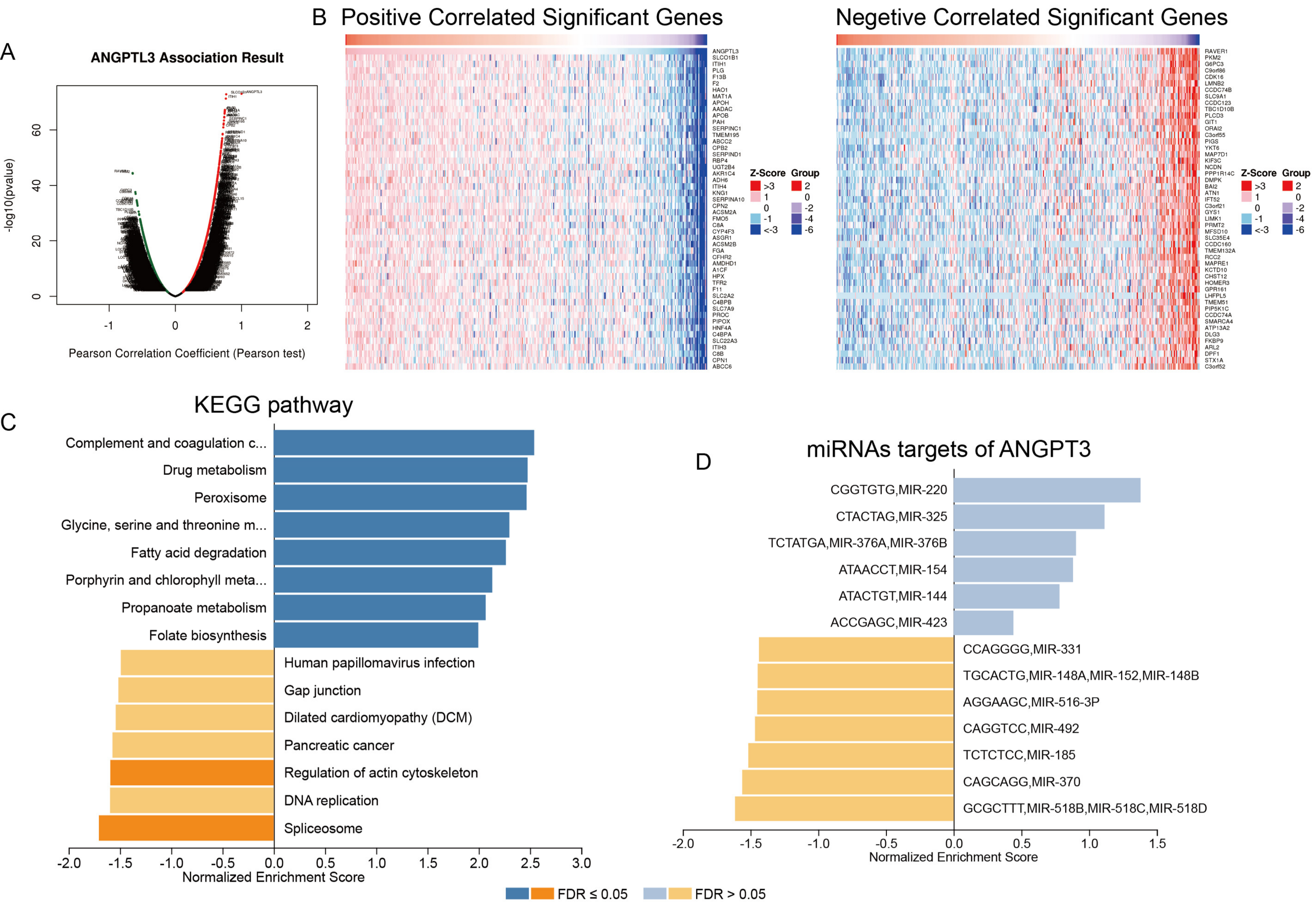
A
ANGPTL3 Association Result
-log10(pvalue)
0
20
40
60
-1
0
1
2
Pearson Correlation Coefficient (Pearson test)
B
Positive Correlated Significant Genes
ANGPTL3 SLCO1B1 ITIH1 PLG F13B F2 HAO1 MAT1A APOH AADAC APOB PAH SERPINC1 TMEM195 ABCC2 CPB2 SERPIND1 RBP4 UGT2B4 AKR1C4 ADH6 ITIH4 KNG1 SERPINA10 CPN2 ACSM2A FMO5 C8A CYP4F3 ASGR1 ACSM2B FGA CFHR2 AMDHD1 A1CF HPX TFR2 F11 SLC2A2 C4BPB SLC7A9 PROC PIPOX HNF4A C4BPA SLC22A3 ITIH3 C8B CPN1 ABCC6
Z-Score
>3
1
0
-1
<-3
Group
2
0
-2
-4
-6
Negetive Correlated Significant Genes
RAVER1 PKM2 G6PC3 C9orf86 CDK16 LMNB2 CCDC74B SLC9A1 CCDC123 TBC1D10B PLCD3 GIT1 ORAI2 C3orf55 PIGS YKT6 MAP7D1 KIF3C NCDN PPP1R14C DMPK BAI2 ATN1 IFT52 C3orf21 GYS1 LIMK1 PRMT2 MFSD10 SLC35E4 CDC160 TMEM132A RCC2 MAPRE1 KCTD10 CHST12 HOMER3 GPR161 LHFPL5 TMEM51 PIP5K1C CCDC74A SMARCA4 ATP13A2 DLG3 FKBP9 ARL2 DPF1 STX1A C3orf52
Z-Score
>3
1
0
-1
<-3
Group
2
0
-2
-4
-6
C
KEGG pathway
Complement and coagulation c...
Drug metabolism
Peroxisome
Glycine, serine and threonine m...
Fatty acid degradation
Porphyrin and chlorophyll meta...
Propanoate metabolism
Folate biosynthesis
Human papillomavirus infection
Gap junction
Dilated cardiomyopathy (DCM)
Pancreatic cancer
Regulation of actin cytoskeleton
DNA replication
Spliceosome
-2.0 -1.5 -1.0 -0.5 0.0 0.5 1.0 1.5 2.0 2.5 3.0
Normalized Enrichment Score
D
miRNAs targets of ANGPT3
CGGTGTG,MIR-220
CTACTAG,MIR-325
TCTATGA,MIR-376A,MIR-376B
ATAACCT,MIR-154
ATACTGT,MIR-144
ACCGAGC,MIR-423
CCAGGGG,MIR-331
TGCACTG,MIR-148A,MIR-152,MIR-148B
AGGAAGC,MIR-516-3P
CAGGTCC,MIR-492
TCTCTCC,MIR-185
CAGCAGG,MIR-370
GCGCTTT,MIR-518B,MIR-518C,MIR-518D
-2.0 -1.5 -1.0 -0.5 0.0 0.5 1.0 1.5
Normalized Enrichment Score
FDR ≤ 0.05
FDR > 0.05
