# Supplementary material for: The Role of ANGPTL Gene Family Members in Hepatocellular Carcinoma
Source: Dis Markers. 2022 Jun 1;2022:1844352. doi: 10.1155/2022/1844352 (PMC9177307; doi:10.1155/2022/1844352)
Supplement: Supplementary Materials — Table S1: differential expression of ANGPTL family members in HCC tissue in HCCDB database. Table S2: incidence rate of ANGPTL mutation in cBioPortal database. Table S3: the mutation incidence rate and copy changes of ANGPTL family genes and nearby genes. Figure S1: the mRNA expression levels of ANGPTL2 (A), ANGPTL3 (B), ANGPTL4 (C), ANGPTL5 (D), ANGPTL6 (E), ANGPTL7 (F), and ANGPTL8 (G), respectively, in several common cancer cell lines in the CCLE database. Figure S2: boxplot showing the difference in expression of ANGPTL mRNA levels in hepatocellular carcinoma cell lines versus other cancer cell lines. Figure S3: violin plot showing the relative expression of ANGPTLs in UCSC database in HCC samples and normal tissue samples. Panels (A)–(F) represent the expression of ANGPTL2, ANGPTL4, ANGPTL5, ANGPTL6, ANGPTL7, and ANGPTL8 mRNAs in HCC samples relative to normal samples based on the TCGA database. LIHC: abbreviation for hepatocellular carcinoma of the liver in the TCGA database. Figure S4: expression levels of ANGPTL2 (A), ANGPTL3 (B), ANGPTL4 (C), ANGPTL5 (D), ANGPTL6 (E), and ANGPTL7 (F) mRNAs in different HCC datasets in the HCCDB database were analyzed. Red: HCC samples; blue: adjacent normal tissue samples; cyan: cirrhotic samples; orange: healthy samples. Figure S5: correlation between ANGPTL gene family and sex-based on TCGA database. Panels (A)–(F) represent ANGPTL1, ANGPTL2, ANGPTL3, ANGPTL4, ANGPTL5, and ANGPTL6, respectively. Figure S6: analysis of ANGPTL DNA methylation levels in HCC tumor tissues versus normal tissues based on TCGA database. Panels (A)–(H) represent ANGPTL1, ANGPTL2, ANGPTL3, ANGPTL4, ANGPTL5, ANGPTL6, ANGPTL7, and ANGPTL8 DNA methylation expressions, respectively. Figure S7: analysis of the correlation between ANGPTL DNA methylation and clinical proportional characteristics by gender. Panels (A)–(H) represent the association of ANGPTL1, ANGPTL2, ANGPTL3, ANGPTL4, ANGPTL5, ANGPTL6, ANGPTL7, and ANGPTL8 DNA methylation with gender, re [file 1844352.f1.zip › Figure S13.pdf]

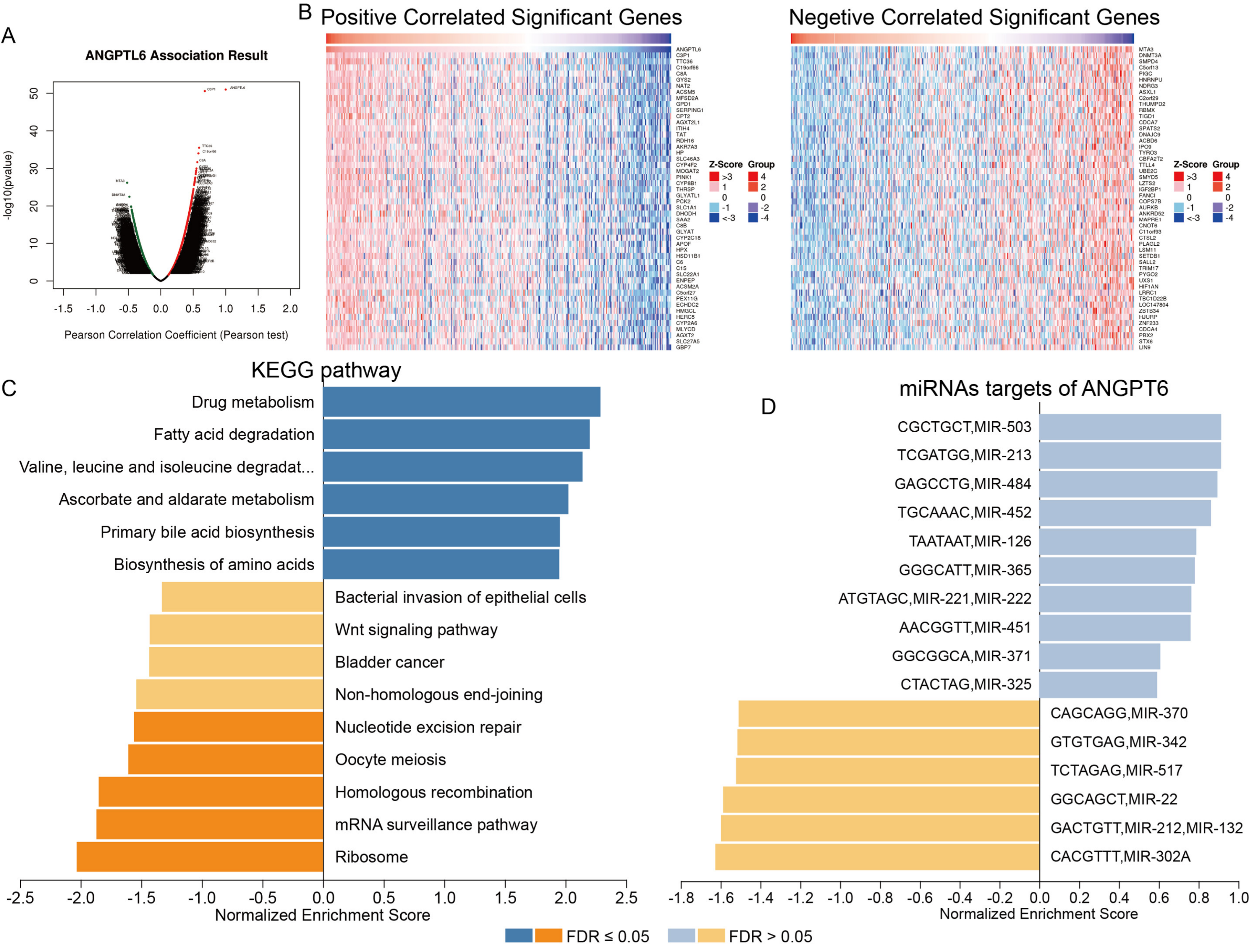
A
ANGPTL6 Association Result
-log10(pvalue)
Pearson Correlation Coefficient (Pearson test)
-1.5 -1.0 -0.5 0.0 0.5 1.0 1.5 2.0
0 10 20 30 40 50
B
Positive Correlated Significant Genes
ANGPTL6
C3P1
TTC36
C19orf66
C8A
GYS2
NAT2
ACSM5
MFSD2A
GPD1
SERPING1
CPT2
AGXT2L1
ITIH4
TAT
RDH16
AKR7A3
HP
SLC46A3
CYP4F2
MOGAT2
PINK1
CYP8B1
THRSP
GLYATL1
PCK2
SLC1A1
DHODH
SAA2
C8B
GLYAT
CYP2C18
APOF
HPX
HSD11B1
C6
C1S
SLC22A1
ENPEP
ACSM2A
C5orf27
PEX11G
ECHDC2
HMGCL
HERC5
CYP2A6
MLYCD
AGXT2
SLC27A5
GBP7
Z-Score
>3
1
0
-1
<-3
Group
4
2
0
-2
-4
Negetive Correlated Significant Genes
MTA3
DNMT3A
SMPD4
C5orf13
PIGC
HNRNPU
NDRG3
ASXL1
C2orf29
THUMPD2
RBMX
TIGD1
CDCA7
SPATS2
DNAJC9
ACBD6
IPO9
TYRO3
CBFA2T2
TTLL4
UBE2C
SMYD5
LZTS2
IGF2BP1
FANCI
COPS7B
AURKB
ANKRD52
MAPRE1
CNOT6
C11orf93
CTSL2
PLAGL2
LSM11
SETDB1
SALL2
TRIM17
PYGO2
UXS1
HIF1AN
LRRC1
TBC1D22B
LOC147804
ZBTB34
HJURP
ZNF233
CDCA4
PBX2
STX6
LIN9
Z-Score
>3
1
0
-1
<-3
Group
4
2
0
-2
-4
C
KEGG pathway
Drug metabolism
Fatty acid degradation
Valine, leucine and isoleucine degradat...
Ascorbate and aldarate metabolism
Primary bile acid biosynthesis
Biosynthesis of amino acids
Bacterial invasion of epithelial cells
Wnt signaling pathway
Bladder cancer
Non-homologous end-joining
Nucleotide excision repair
Oocyte meiosis
Homologous recombination
mRNA surveillance pathway
Ribosome
-2.5 -2.0 -1.5 -1.0 -0.5 0.0 0.5 1.0 1.5 2.0 2.5
Normalized Enrichment Score
D
miRNAs targets of ANGPT6
CGCTGCT,MIR-503
TCGATGG,MIR-213
GAGCCTG,MIR-484
TGCAAAC,MIR-452
TAATAAT,MIR-126
GGGCATT,MIR-365
ATGTAGC,MIR-221,MIR-222
AACGGTT,MIR-451
GGCGGCA,MIR-371
CTACTAG,MIR-325
CAGCAGG,MIR-370
GTGTGAG,MIR-342
TCTAGAG,MIR-517
GGCAGCT,MIR-22
GACTGTT,MIR-212,MIR-132
CACGTTT,MIR-302A
-1.8 -1.6 -1.4 -1.2 -1.0 -0.8 -0.6 -0.4 -0.2 0.0 0.2 0.4 0.6 0.8 1.0
Normalized Enrichment Score
FDR ≤ 0.05
FDR > 0.05
