# Supplementary material for: The Role of ANGPTL Gene Family Members in Hepatocellular Carcinoma
Source: Dis Markers. 2022 Jun 1;2022:1844352. doi: 10.1155/2022/1844352 (PMC9177307; doi:10.1155/2022/1844352)
Supplement: Supplementary Materials — Table S1: differential expression of ANGPTL family members in HCC tissue in HCCDB database. Table S2: incidence rate of ANGPTL mutation in cBioPortal database. Table S3: the mutation incidence rate and copy changes of ANGPTL family genes and nearby genes. Figure S1: the mRNA expression levels of ANGPTL2 (A), ANGPTL3 (B), ANGPTL4 (C), ANGPTL5 (D), ANGPTL6 (E), ANGPTL7 (F), and ANGPTL8 (G), respectively, in several common cancer cell lines in the CCLE database. Figure S2: boxplot showing the difference in expression of ANGPTL mRNA levels in hepatocellular carcinoma cell lines versus other cancer cell lines. Figure S3: violin plot showing the relative expression of ANGPTLs in UCSC database in HCC samples and normal tissue samples. Panels (A)–(F) represent the expression of ANGPTL2, ANGPTL4, ANGPTL5, ANGPTL6, ANGPTL7, and ANGPTL8 mRNAs in HCC samples relative to normal samples based on the TCGA database. LIHC: abbreviation for hepatocellular carcinoma of the liver in the TCGA database. Figure S4: expression levels of ANGPTL2 (A), ANGPTL3 (B), ANGPTL4 (C), ANGPTL5 (D), ANGPTL6 (E), and ANGPTL7 (F) mRNAs in different HCC datasets in the HCCDB database were analyzed. Red: HCC samples; blue: adjacent normal tissue samples; cyan: cirrhotic samples; orange: healthy samples. Figure S5: correlation between ANGPTL gene family and sex-based on TCGA database. Panels (A)–(F) represent ANGPTL1, ANGPTL2, ANGPTL3, ANGPTL4, ANGPTL5, and ANGPTL6, respectively. Figure S6: analysis of ANGPTL DNA methylation levels in HCC tumor tissues versus normal tissues based on TCGA database. Panels (A)–(H) represent ANGPTL1, ANGPTL2, ANGPTL3, ANGPTL4, ANGPTL5, ANGPTL6, ANGPTL7, and ANGPTL8 DNA methylation expressions, respectively. Figure S7: analysis of the correlation between ANGPTL DNA methylation and clinical proportional characteristics by gender. Panels (A)–(H) represent the association of ANGPTL1, ANGPTL2, ANGPTL3, ANGPTL4, ANGPTL5, ANGPTL6, ANGPTL7, and ANGPTL8 DNA methylation with gender, re [file 1844352.f1.zip › Figure S14.pdf]

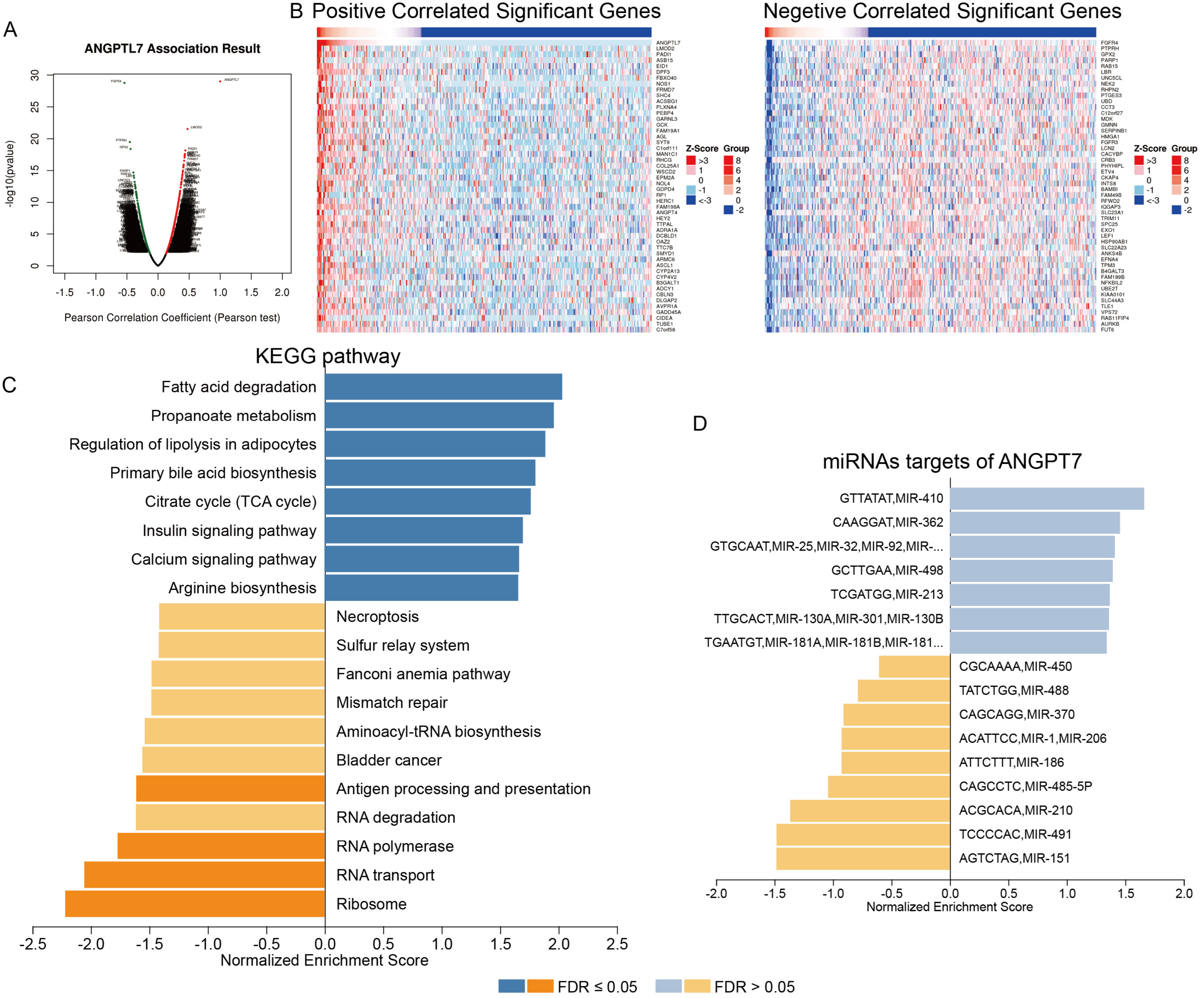
A
ANGPTL7 Association Result
-log10(pvalue)
Pearson Correlation Coefficient (Pearson test)
FGFR4
ANGPTL7
LMOD2
PTPRH
GPX2
B Positive Correlated Significant Genes
ANGPTL7
LMOD2
PADI1
ASB15
EID1
DPF3
FBXO40
NOS1
FRMD7
SHC4
ACSBG1
PLXNA4
PEBP4
GARNL3
GCK
FAM19A1
AGL
SYT9
C1orf111
MAN1C1
RHCG
COL25A1
WSCD2
EPM2A
NOL4
GDPD4
RP1
HERC1
FAM198A
ANGPT4
HEY2
TTPAL
ADRA1A
DCBLD1
OAZ2
TTC7B
SMYD1
ARMC6
ASCL1
CYP2A13
CYP4V2
B3GALT1
ADCY1
CBLN3
DLGAP2
AVPR1A
GADD45A
CIDEA
TUBE1
C7orf58
Z-Score
>3
1
0
-1
<-3
Group
8
6
4
2
0
-2
Negetive Correlated Significant Genes
FGFR4
PTPRH
GPX2
PARP1
RAB15
LBR
UNC5CL
NEK2
RHPN2
PTGES3
UBD
CCT3
C12orf27
MDK
GMNN
SERPINB1
HMGA1
FGFR3
LCN2
CACYBP
CRB3
PHYHIPL
ETV4
CKAP4
INTS8
BAMBI
FAM49B
RFWD2
IQGAP3
SLC23A1
TRIM11
SPC25
EXO1
LEF1
HSP90AB1
SLC22A23
ANKS4B
EFNA4
TPM3
B4GALT3
FAM189B
NFKBIL2
UBE2T
KIAA0101
SLC44A3
TLE1
VPS72
RAB11FIP4
AURKB
FUT6
Z-Score
>3
1
0
-1
<-3
Group
8
6
4
2
0
-2
KEGG pathway
C
Fatty acid degradation
Propanoate metabolism
Regulation of lipolysis in adipocytes
Primary bile acid biosynthesis
Citrate cycle (TCA cycle)
Insulin signaling pathway
Calcium signaling pathway
Arginine biosynthesis
Necroptosis
Sulfur relay system
Fanconi anemia pathway
Mismatch repair
Aminoacyl-tRNA biosynthesis
Bladder cancer
Antigen processing and presentation
RNA degradation
RNA polymerase
RNA transport
Ribosome
-2.5 -2.0 -1.5 -1.0 -0.5 0.0 0.5 1.0 1.5 2.0 2.5
Normalized Enrichment Score
D
miRNAs targets of ANGPT7
GTTATAT,MIR-410
CAAGGAT,MIR-362
GTGCAAT,MIR-25,MIR-32,MIR-92,MIR-...
GCTTGAA,MIR-498
TCGATGG,MIR-213
TTGCACT,MIR-130A,MIR-301,MIR-130B
TGAATGT,MIR-181A,MIR-181B,MIR-181...
CGCAAAA,MIR-450
TATCTGG,MIR-488
CAGCAGG,MIR-370
ACATTCC,MIR-1,MIR-206
ATTCTTT,MIR-186
CAGCCTC,MIR-485-5P
ACGCACA,MIR-210
TCCCCAC,MIR-491
AGTCTAG,MIR-151
-2.0 -1.5 -1.0 -0.5 0.0 0.5 1.0 1.5 2.0
Normalized Enrichment Score
FDR ≤ 0.05
FDR > 0.05
