# Supplementary material for: The Role of ANGPTL Gene Family Members in Hepatocellular Carcinoma
Source: Dis Markers. 2022 Jun 1;2022:1844352. doi: 10.1155/2022/1844352 (PMC9177307; doi:10.1155/2022/1844352)
Supplement: Supplementary Materials — Table S1: differential expression of ANGPTL family members in HCC tissue in HCCDB database. Table S2: incidence rate of ANGPTL mutation in cBioPortal database. Table S3: the mutation incidence rate and copy changes of ANGPTL family genes and nearby genes. Figure S1: the mRNA expression levels of ANGPTL2 (A), ANGPTL3 (B), ANGPTL4 (C), ANGPTL5 (D), ANGPTL6 (E), ANGPTL7 (F), and ANGPTL8 (G), respectively, in several common cancer cell lines in the CCLE database. Figure S2: boxplot showing the difference in expression of ANGPTL mRNA levels in hepatocellular carcinoma cell lines versus other cancer cell lines. Figure S3: violin plot showing the relative expression of ANGPTLs in UCSC database in HCC samples and normal tissue samples. Panels (A)–(F) represent the expression of ANGPTL2, ANGPTL4, ANGPTL5, ANGPTL6, ANGPTL7, and ANGPTL8 mRNAs in HCC samples relative to normal samples based on the TCGA database. LIHC: abbreviation for hepatocellular carcinoma of the liver in the TCGA database. Figure S4: expression levels of ANGPTL2 (A), ANGPTL3 (B), ANGPTL4 (C), ANGPTL5 (D), ANGPTL6 (E), and ANGPTL7 (F) mRNAs in different HCC datasets in the HCCDB database were analyzed. Red: HCC samples; blue: adjacent normal tissue samples; cyan: cirrhotic samples; orange: healthy samples. Figure S5: correlation between ANGPTL gene family and sex-based on TCGA database. Panels (A)–(F) represent ANGPTL1, ANGPTL2, ANGPTL3, ANGPTL4, ANGPTL5, and ANGPTL6, respectively. Figure S6: analysis of ANGPTL DNA methylation levels in HCC tumor tissues versus normal tissues based on TCGA database. Panels (A)–(H) represent ANGPTL1, ANGPTL2, ANGPTL3, ANGPTL4, ANGPTL5, ANGPTL6, ANGPTL7, and ANGPTL8 DNA methylation expressions, respectively. Figure S7: analysis of the correlation between ANGPTL DNA methylation and clinical proportional characteristics by gender. Panels (A)–(H) represent the association of ANGPTL1, ANGPTL2, ANGPTL3, ANGPTL4, ANGPTL5, ANGPTL6, ANGPTL7, and ANGPTL8 DNA methylation with gender, re [file 1844352.f1.zip › Figure S9.pdf]

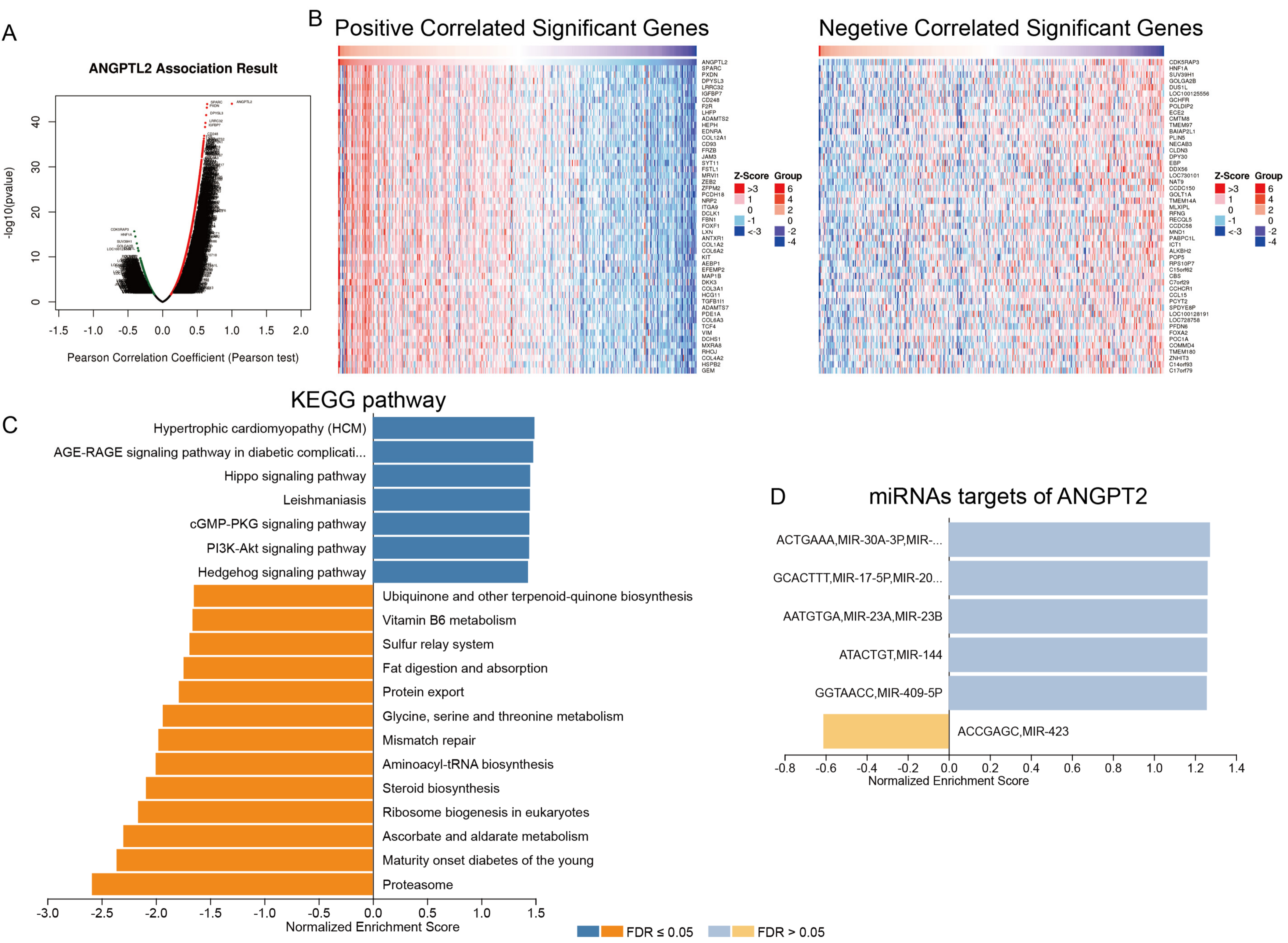
A
ANGPTL2 Association Result
-log10(pvalue)
Pearson Correlation Coefficient (Pearson test)
-1.5 -1.0 -0.5 0.0 0.5 1.0 1.5 2.0
0 10 20 30 40
B
Positive Correlated Significant Genes
ANGPTL2 SPARC PXDN DPYSL3 LRRC32 IGFBP7 CD248 F2R LHFP ADAMTS2 HEPH EDNRA COL12A1 CD93 FRZB JAM3 SYT11 FSTL1 MRVI1 ZEB2 ZFPM2 PCDH18 NRP2 ITGA9 DCLK1 FBN1 FOXF1 LXN ANTXR1 COL1A2 COL6A2 KIT AEBP1 EFEMP2 MAP1B DKK3 COL3A1 HCG11 TGFB1I1 ADAMTS7 PDE1A COL6A3 TCF4 VIM DCHS1 MXRA8 RHOJ COL4A2 HSPB2 GEM
Z-Score >3 1 0 -1 <-3
Group 6 4 2 0 -2 -4
Negetive Correlated Significant Genes
CDK5RAP3 HNF1A SUV39H1 GOLGA2B DUS1L LOC100125556 GCHFR POLDIP2 ECE2 CMTM8 TMEM97 BAIAP2L1 PLIN5 NECAB3 CLDN3 DPY30 EBP DDX56 LOC730101 NAT9 CCDC150 GOLT1A TMEM14A MLXIPL RFNG RECQL5 CCDC58 MND1 PABPC1L ICT1 ALKBH2 POP5 RPS10P7 C15orf62 CBS C7orf29 CCHCR1 CCL15 PCYT2 SPDYE8P LOC100128191 LOC728758 PFDN6 FOXA2 POC1A COMMD4 TMEM180 ZNHIT3 C14orf93 C17orf79
Z-Score >3 1 0 -1 <-3
Group 6 4 2 0 -2 -4
KEGG pathway
C
Hypertrophic cardiomyopathy (HCM)
AGE-RAGE signaling pathway in diabetic complicati...
Hippo signaling pathway
Leishmaniasis
cGMP-PKG signaling pathway
PI3K-Akt signaling pathway
Hedgehog signaling pathway
Ubiquinone and other terpenoid-quinone biosynthesis
Vitamin B6 metabolism
Sulfur relay system
Fat digestion and absorption
Protein export
Glycine, serine and threonine metabolism
Mismatch repair
Aminoacyl-tRNA biosynthesis
Steroid biosynthesis
Ribosome biogenesis in eukaryotes
Ascorbate and aldarate metabolism
Maturity onset diabetes of the young
Proteasome
-3.0 -2.5 -2.0 -1.5 -1.0 -0.5 0.0 0.5 1.0 1.5
Normalized Enrichment Score
D
miRNAs targets of ANGPT2
ACTGAAA,MIR-30A-3P,MIR-...
GCACTTT,MIR-17-5P,MIR-20...
AATGTGA,MIR-23A,MIR-23B
ATACTGT,MIR-144
GGTAACC,MIR-409-5P
ACCGAGC,MIR-423
-0.8 -0.6 -0.4 -0.2 0.0 0.2 0.4 0.6 0.8 1.0 1.2 1.4
Normalized Enrichment Score
FDR ≤ 0.05
FDR > 0.05
