# Supplementary material for: The Role of ANGPTL Gene Family Members in Hepatocellular Carcinoma
Source: Dis Markers. 2022 Jun 1;2022:1844352. doi: 10.1155/2022/1844352 (PMC9177307; doi:10.1155/2022/1844352)
Supplement: Supplementary Materials — Table S1: differential expression of ANGPTL family members in HCC tissue in HCCDB database. Table S2: incidence rate of ANGPTL mutation in cBioPortal database. Table S3: the mutation incidence rate and copy changes of ANGPTL family genes and nearby genes. Figure S1: the mRNA expression levels of ANGPTL2 (A), ANGPTL3 (B), ANGPTL4 (C), ANGPTL5 (D), ANGPTL6 (E), ANGPTL7 (F), and ANGPTL8 (G), respectively, in several common cancer cell lines in the CCLE database. Figure S2: boxplot showing the difference in expression of ANGPTL mRNA levels in hepatocellular carcinoma cell lines versus other cancer cell lines. Figure S3: violin plot showing the relative expression of ANGPTLs in UCSC database in HCC samples and normal tissue samples. Panels (A)–(F) represent the expression of ANGPTL2, ANGPTL4, ANGPTL5, ANGPTL6, ANGPTL7, and ANGPTL8 mRNAs in HCC samples relative to normal samples based on the TCGA database. LIHC: abbreviation for hepatocellular carcinoma of the liver in the TCGA database. Figure S4: expression levels of ANGPTL2 (A), ANGPTL3 (B), ANGPTL4 (C), ANGPTL5 (D), ANGPTL6 (E), and ANGPTL7 (F) mRNAs in different HCC datasets in the HCCDB database were analyzed. Red: HCC samples; blue: adjacent normal tissue samples; cyan: cirrhotic samples; orange: healthy samples. Figure S5: correlation between ANGPTL gene family and sex-based on TCGA database. Panels (A)–(F) represent ANGPTL1, ANGPTL2, ANGPTL3, ANGPTL4, ANGPTL5, and ANGPTL6, respectively. Figure S6: analysis of ANGPTL DNA methylation levels in HCC tumor tissues versus normal tissues based on TCGA database. Panels (A)–(H) represent ANGPTL1, ANGPTL2, ANGPTL3, ANGPTL4, ANGPTL5, ANGPTL6, ANGPTL7, and ANGPTL8 DNA methylation expressions, respectively. Figure S7: analysis of the correlation between ANGPTL DNA methylation and clinical proportional characteristics by gender. Panels (A)–(H) represent the association of ANGPTL1, ANGPTL2, ANGPTL3, ANGPTL4, ANGPTL5, ANGPTL6, ANGPTL7, and ANGPTL8 DNA methylation with gender, re [file 1844352.f1.zip › Table S1.docx]

**Table S1. Differential expression of ANGPTL family members in HCC tissue in HCCDB database.**

| **ANGPTL members** | **HCC database** | **P-value** | **Type** | **Mean** |
| --- | --- | --- | --- | --- |
| ANGPTL1 | HCCDB1 | 8.51E-33 | HCC | 6.495 |
|  |  |  | Adjacent | 8.552 |
|  | HCCDB12 | 1.42E-09 | HCC | 5.347 |
|  |  |  | Adjacent | 6.48 |
|  | HCCDB13 | 7.18E-37 | HCC | 4.429 |
|  |  |  | Adjacent | 5.405 |
|  | HCCDB15 | 2.63E-49 | HCC | 5.767 |
|  |  |  | Adjacent | 8.612 |
|  | HCCDB16 | 1.61E-11 | HCC | 6.343 |
|  |  |  | Adjacent | 7.621 |
|  | HCCDB18 | 1.01E-46 | HCC | 0.9876 |
|  |  |  | Adjacent | 2.115 |
| ANGPTL2 | HCCDB13 | 2.69E-17 | HCC | 5.047 |
|  |  |  | Adjacent | 5.732 |
|  | HCCDB15 | 1.21E-10 | HCC | 8.409 |
|  |  |  | Adjacent | 9.138 |
| ANGPTL3 | HCCDB1 | 8.37E-14 | HCC | 10.97 |
|  |  |  | Adjacent | 12.05 |
|  | HCCDB3 | 5.95E-35 | HCC | 5.602 |
|  |  |  | Adjacent | 8.892 |
|  |  |  | Cirrhotic | 5.662 |
|  |  |  | Healthy | 9.165 |
|  | HCCDB6 | 6.42E-25 | HCC | 9.016 |
|  |  |  | Adjacent | 10.27 |
|  | HCCDB12 | 0.000015 | HCC | 11.91 |
|  |  |  | Adjacent | 12.97 |
|  | HCCDB13 | 1.57E-16 | HCC | 10.43 |
|  |  |  | Adjacent | 11.16 |
|  | HCCDB15 | 1.70E-29 | HCC | 12.26 |
|  |  |  | Adjacent | 13.98 |
|  | HCCDB18 | 3.01E-10 | HCC | 6.92 |
|  |  |  | Adjacent | 7.672 |
| ANGPTL4 | HCCDB1 | 1.48E-15 | HCC | 10.71 |
|  |  |  | Adjacent | 11.9 |
|  | HCCDB3 | 2.54E-28 | HCC | 4.898 |
|  |  |  | Adjacent | 8.031 |
|  |  |  | Cirrhotic | 7.552 |
|  |  |  | Healthy | 6.405 |
|  | HCCDB13 | 3.54E-15 | HCC | 7.917 |
|  |  |  | Adjacent | 8.792 |
|  | HCCDB15 | 4.76E-10 | HCC | 11.01 |
|  |  |  | Adjacent | 12.23 |
|  | HCCDB17 | 9.54E-09 | HCC | 8.784 |
|  |  |  | Adjacent | 9.456 |
|  | HCCDB18 | 0.00000337 | HCC | 4.546 |
|  |  |  | Adjacent | 5.163 |
| ANGPTL6 | HCCDB1 | 9.77E-50 | HCC | 8.912 |
|  |  |  | Adjacent | 11.15 |
|  | HCCDB3 | 1.03E-57 | HCC | 0.1391 |
|  |  |  | Adjacent | 0.8732 |
|  |  |  | Cirrhotic | 0.7051 |
|  |  |  | Healthy | 0.63 |
|  | HCCDB4 | 9.94E-74 | HCC | 6.815 |
|  |  |  | Adjacent | 7.718 |
|  | HCCDB12 | 1.14E-16 | HCC | 9.067 |
|  |  |  | Adjacent | 10.47 |
|  | HCCDB13 | 1.41E-91 | HCC | 4.985 |
|  |  |  | Adjacent | 6.664 |
|  | HCCDB15 | 2.20E-111 | HCC | 5.901 |
|  |  |  | Adjacent | 9.629 |
|  | HCCDB16 | 7.88E-17 | HCC | 7.518 |
|  |  |  | Adjacent | 8.131 |
|  | HCCDB17 | 7.03E-25 | HCC | 7.292 |
|  |  |  | Adjacent | 8.512 |
|  | HCCDB18 | 4.36E-25 | HCC | 3.901 |
|  |  |  | Adjacent | 4.615 |
| ANGPTL8 | HCCDB4 | 3.31E-12 | HCC | 9.366 |
|  |  |  | Adjacent | 8.399 |
